# Consumer Assessment of Healthcare Providers and Systems (CAHPS®) survey of experiences with ambulatory healthcare for Asians and non-Hispanic Whites in the United States

**DOI:** 10.1186/s41687-021-00303-3

**Published:** 2021-03-24

**Authors:** Mohir Ahmedov, Nadereh Pourat, Honghu Liu, Ron D. Hays

**Affiliations:** 1 Health, Nutrition and Population Global Practice, World Bank, Washington, DC, USA; 2 Center for Health Policy Research, University of California, Los Angeles, California USA; 3 School of Dentistry, David Geffen School of Medicine, University of California Los Angeles, Los Angeles, USA; 4 Division of General Internal Medicine & Health Services Research, David Geffen School of Medicine, University of California Los Angeles, 1100 Glendon Avenue, Los Angeles, CA 90024 USA

**Keywords:** CAHPS®, Experiences with care, Asians

## Abstract

**Background:**

Differences in experiences of care reported by Asian Americans (Asians) compared to non-Hispanic Whites (Whites) may be due to lack of measurement invariance.

**Methods:**

We evaluated the three-factor structure and the equivalence of responses to the Consumer Assessment of Healthcare Providers and Systems (CAHPS®) Clinical and Group (CG-CAHPS) Adult Visit Survey 1.0 and compared care experiences of Asians and Whites. Thirteen questions were used to elicit reports about specific aspects of care and two questions assessed overall care perceptions.

This analysis of the CAHPS database included 769 providers and 266,327 respondents. Most surveys (98%) were administered by mail and the rest (2%) by phone. Only 0.5% of the surveys were administered in Spanish. The sample was 64% female, 89% White and 2% Asian, 39% 65 years or older, and 32% were high school graduates or less.

**Results:**

A three-factor model was supported by categorical confirmatory factor analysis using weighted least squares with mean and variance adjustment: confirmatory fit index (CFI) = 0.99 and root mean squared error of approximation (RMSEA) = 0.03). A multi-group configural invariance model also fit the data well: (CFI = 0.993, RMSEA = 0.031). Regression models indicated that Asians reported worse *access*, lower scores on *office staff courtesy and helpfulness* and *rating their doctors* and were less likely *to recommend their doctors* to family/friends than did Whites.

**Conclusions:**

Use of the CG-CAHPS Adult Visit Survey 1.0 to assess perceptions of care by Asians and Whites is supported. Quality improvement efforts are needed to address worse experiences of care among Asians in the United States.

**Supplementary Information:**

The online version contains supplementary material available at 10.1186/s41687-021-00303-3.

## Background

Patient evaluations of care are used by health plans, physician groups, hospitals, and other health care providers to inform patients about their options and improve quality of care [1–4]. Several studies have found racial/ethnic disparities in patient experiences with care. For example, Snyder and colleagues [5] found that Asian Americans reported worst self-reported access to care (wait times, reaching the doctor’s office via phone) than all racial/ethnic groups. Phillips et al. [6] analyzed the 1996 Medical Expenditure Panel Survey data and found that African Americans, Hispanics, Asian Americans and Pacific Islanders were less satisfied with care than non-Hispanic Whites, and Asian Americans were the most dissatisfied. Asian Americans were also less satisfied with care in the 1998 National Research Corporation Healthcare Market Guide® survey [7]. Morales et al. [8] analyzed Consumer Assessment of Healthcare Providers and Systems (CAHPS®) Health Plan 1.0 survey data collected from 54 commercial and 34 Medicaid health plans and found that most minorities reported experiences like non-Hispanic Whites, except for Asian Americans, who expressed worse perceptions of care. Weech-Maldonado and colleagues [9] found that racial/ethnic minorities fared worse than non-Hispanic Whites on the CAHPS Health Plan 2.0 survey. Several other studies have confirmed worse experiences with care for Asian Americans [10, 11].

Comparisons between Asian Americans and non-Hispanic Whites requires measurement equivalence between the two racial/ethnic groups. Dyer and colleagues [12] found support for three factors for the CG-CAHPS Adult Visit 1.0 Survey: *access to care*, *physician communication* and *office staff courtesy and helpfulness*. However, the authors used a CFA model that assumes continuous measures, while the appropriate model for the analysis would be categorical factor analysis. The authors also included in the analysis only respondents that answered all CG-CAHPS core questions (21,318 out of 103,442 respondents). Factor analyses of the CAHPS Medicare survey provided support for measurement equivalence between non-Hispanic Whites (*n* = 1,326,410) and Asians (*n* = 40,672) for the composites (communication, access, customer service) and global ratings [13].

This paper evaluates measurement equivalence on the CAHPS clinician and group adult visit survey 1.0 composites (access to care, physician communication, and office staff courtesy and respect) between non-Hispanic Whites and Asians. Prior to evaluating measurement equivalence, we assess whether the three-factor structure of the CG-CAHPS items observed by Dyer et al. [12] is replicated using categorical confirmatory factor analysis in this dataset. This is necessary to establish before evaluating measurement equivalence of the CAHPS survey for non-Hispanic Whites and Asians. Finally, we compare reports and ratings of care between these two race/ethnic subgroups.

## Methods

### Sample

The dataset used in this analysis includes 769 providers and 266,327 respondents. Most surveys (98%) were administered by mail and the rest (2%) by phone. Only 0.5% of the surveys were administered in Spanish. Sample characteristics and data missingness are provided in Table 1. Respondents were predominantly non-Hispanic White and educated.

**Table 1:** Sample demographic information, CAHPS clinician and group adult visit survey 1.0

	Percent	Frequency
Gender		
Male	36.15	94,068
Female	63.85	160,236
Total	100.00	260,236
Missing values		6091
Age		
18–24	2.83	7364
25–34	7.96	20,733
35–44	9.29	24,207
45–54	16.93	44,118
55–64	24.51	63,872
65–74	21.44	55,872
75+	17.06	44,455
Total	100.00	260,621
Missing values		5706
**Education**		
8th grade or less	2.47	6329
Less than HS grad	4.62	11,845
High School Graduate (includes Graduate Equivalency Degree)	25.15	64,475
Some college	30.23	77,511
4 year graduate	17.43	44,681
More than 4 years	20.10	51,542
Total	100.00	256,383
Missing values		9944
Race/Ethnicity		
White	89.49	229,899
African-American	4.30	11,057
Asian	2.16	5545
Native Hawaiian/Pacific Islander	0.13	338
American Indian/Native Alaskan	0.32	839
Other	2.17	5575
Multi-racial	1.42	3652
Total	100.00	256,885
Missing values		9442
Hispanic/Latino origin or descent		
Yes	4.95	12,475
No	95.05	239,722
Total	100.00	252,197
Missing values		14,130
Health status		
Excellent	12.60	32,747
Very Good	34.41	89,428
Good	33.99	88,353
Fair	15.25	39,625
Poor	3.75	9752
Total	100.00	259,905
Missing values		6422
Practice regions		
Midwest	52.48	139,775
Northeast	22.93	61,056
South	10.08	26,843
West	14.51	38,653
Total	100.00	266,327
Missing values		6422

Percentages do not always add up to 100.00 because of rounding

#### Instrument

The C-G CAHPS Adult Visit Survey 1.0 asks patients about their most recent visit to a doctor (a primary care doctor or a specialist). Thirteen questions are used to create three reporting composites – *access to care* (6 questions), *physician communication* (5 questions) and *office staff courtesy and helpfulness* (2 questions) and there are two global rating questions. Table 2 shows the 15 questions used to elicit reports and ratings of care and their response options.

**Table 2:** CAHPS clinician and group adult visit survey 1.0 reports and ratings of care items

**Physician communication and global ratings**	
1. During your most recent visit, did this doctor explain things in a way that was easy to understand?	
2. During your most recent visit, did this doctor listen carefully to you?	
3. During your most recent visit, did this doctor give you easy to understand instructions about taking care of these health problems or concerns?	
4. During your most recent visit, did this doctor seem to know the important information about your medical history?	
5. During your most recent visit, did this doctor show respect for what you had to say?	
6. During your most recent visit, did this doctor spend enough time with you?	
7. Using any number from 0 to 10, where 0 is the worst doctor possible and 10 is the best doctor possible, what number would you use to rate this doctor?	
8. Would you recommend this doctor’s office to your family and friends?	
**Access to care**	
1. In the last 12 months, when you phoned this doctor’s office to get an appointment for care you needed right away, how often did you get an appointment as soon as you thought you needed?	
2. In the last 12 months, when you made an appointment for a check-up or routine care with this doctor, how often did you get an appointment as soon as you thought you needed?	
3. In the last 12 months, when you phoned this doctor’s office during regular office hours, how often did you get an answer to your medical question that same day?	
4. In the last 12 months, when you phoned this doctor’s office after regular office hours, how often did you get an answer to your medical question as soon as you needed?	
5. Wait time includes time spent in the waiting room and exam room. In the last 12 months, how often did you see this doctor within 15 min of your appointment time?	
**Office staff courtesy and helpfulness**	
1. During your most recent visit, were clerks and receptionists at this doctor’s office as helpful as you thought they should be?	
2. During your most recent visit, did clerks and receptionists at this doctor’s office treat you with courtesy and respect?	

First six items in *physician communication and global ratings,* both items in *office staff courtesy and helpfulness* and the *recommending doctor to friends/family* question use a three-response option scale (*Yes, definitely; Yes, somewhat; No*). *Access to care* composite items use a four-response option scale (*Never, Sometimes, Usually, Always*). The *doctor rating* question uses a 0–10 response scale

The *access to care* composite items asks about timeliness of care, timeliness of responses to medical questions over the phone and waiting times for appointments during the last 12 months using four-category response options (*Always, Usually, Sometimes, Never*). The *physician communication* composite questions refer to the most recent visit and ask whether the doctor listened carefully, gave clear instructions, showed respect, spent enough time with and seemed knowledgeable about respondent’s medical history using a three-category response scale (*Yes, definitely; Yes, somewhat; No*). The *office staff courtesy and helpfulness* composite questions refer to the most recent visit and ask whether clerks and receptionists were helpful and treated the respondent with courtesy and respect using the same three-category response scale used for physician communication. The global rating items ask respondents *(1)* to rate their doctor on a 0–10 rating scale where 0 is the worst possible doctor and 10 is the best doctor and *(2)* whether they would recommend their doctor to family and friends using the three-category response scale (*Yes, definitely; Yes, somewhat; No*).

Two questions in the CAHPS surveys ask about race/ethnicity. The first question asks if the respondent is of Hispanic or Latino origin. The next question asks whether the respondent is: 1) White, 2) Black or African American, 3) Asian, 4) Native Hawaiian or Pacific Islander, 5) American Indian or Alaska Native, or 6) other. Like the US Decennial Census, those respondents confirming Hispanic or Latino origin were coded as Hispanic. Those who reported no Hispanic origin or had a missing value were coded in accordance with their self-reported race. The resulting race/ethnic categories were Hispanic, White, Black/African American, Asian and other: Native Hawaiians or Pacific Islanders, American Indians, Alaska Native and other races were coded into the *other* group. The questionnaire also assessed patient gender, education, age, and health status (Table 1).

### Analysis plan

The evaluation of factor structure and measurement invariance was limited to the White and Asian subgroups. The assessment of differences in care experiences for these two subgroups was performed in a related rather than isolated model environment using the full sample to improve model fitting.

#### Evaluation of factor structure

We assess a three-factor structure (*access to care*, *physician communication* and *office staff courtesy and helpfulness*) shown in the online supplemental material (Figure S1). We evaluate whether a multi-level model was necessary using an intraclass correlation threshold of 0.10 or higher [14]. We conducted categorical confirmatory factor analysis with Mplus 7 using the robust weighted least squares estimation procedure, the weighted least square mean and variance adjusted estimation (WLSMV). We used theta parameterization and full information maximum likelihood estimation. Theta parameterization is a model specification method that considers variance differences across groups [15]. We evaluated model fit using a parsimony correction index root mean squared error of approximation (RMSEA) and the comparative fit index (CFI). RMSEA values of 0.05 or less are considered a close fit. CFI of 0.95 or greater are considered acceptable fit [16]. We used a standardized factor loading of 0.40 or greater as the threshold for the appropriateness of the item for the proposed factor [17]. We estimated correlations among factors, with very high correlations (0.80 or above) implying that factors are redundant.

#### Evaluation of measurement invariance

We fit multiple group confirmatory factor analysis (MG CFA) models to assess measurement invariance between Asians and Whites. Configural invariance confirms that the number of factors and the pattern of indicator-factor loadings are identical across the groups. Metric and scalar invariance confirm equality of factor loadings, and factors loadings and intercepts, respectively [18].

In the first step, we performed single group CFA separately in the White and Asian subgroups to confirm that the measurement model had an acceptable fit in the two groups. Model fit indices and parameter estimates for the two groups were evaluated. In the second step, we conducted a test of configural invariance to explore whether the pattern of free factor loadings and thresholds were similar across groups. Lack of configural invariance implies that different latent variables in the two groups may have been measured. In the third step, we first constrained factor loadings to equality in the two groups to conduct a metric invariance test. Evidence of metric invariance implies that latent variables are related to survey items the same way across groups. That is, one-unit change in item score translates to the same unit change in estimated latent variable score in the groups. Once the metric invariance test results show an acceptable model fit, a new constraint – equality of thresholds across groups – was added to test for scalar invariance. Evidence of scalar invariance implies that individuals with the same score on a latent variable are likely to have the same score on survey items corresponding to the latent variable. Thus, score means across groups can be compared validly.

We performed two versions of a metric invariance test. In the first version, we followed an approach suggested by Brown [18], where we fixed factor means to 0 in both groups and factor variances to 1 in one group and placed no constraints in the other. This model produced a difference of 12 constraints (difference in degrees of freedom) between the configural and metric models, while we placed a constraint on 15 parameters (15 factor loadings). In order to address this issue, we fixed factor means to 0 and factor variance to 1 in both groups and obtained a difference in degrees of freedom corresponding to the number of constrained parameters.

We also performed two versions of scalar invariance testing. In the first approach (proposed in [18]), we fixed factor means to 0 and factor variances to 1 in one group and set both parameters free in the second group. The test results showed a difference in degrees of freedom between the two models to be 33, while we placed constraints on 36 parameters. In the second approach, we fixed factor means to 0 and factor variances to 1 in both groups, which yielded a difference in degrees of freedom of 36.

In nested models, chi square values are generally expected to be higher for the more constrained models compared to the less constrained models. In our analysis, chi square values in the metric models were consistently lower than those in the configural models. Chi square values from nested models are considered not to be directly comparable when the WLSMV estimation is used. This places limitation on evaluation of a model fit, as many practical fit indices are derived from chi square values. To confirm the model findings, we also estimated models with an MLR estimation. The MLR based models also produced lower chi square values in the metric models compared to those in the configural models. Therefore, in the final step, we run the models with the ML estimation. The chi square values in more constrained models were higher than those in less constrained models when using the ML estimation, thus permitting comparison of model fit indices across models. CFI values in nested models using the WLSMV and MLR estimations may not be directly comparable [19].

#### Evaluation of racial-ethnic differences in patient experiences with care

First, an ordinary least squares (OLS) model was run with the main effects in the model, adjusting for practice site using the cluster option in STATA 14. Adjusted scores (means) for Asians and Whites for composites and global ratings were estimated using recycled predictions in STATA 14. Recycled predictions were obtained from regression models and used to understand marginal effects of independent variables on a dependent variable. Independent variables other than the one of primary interest were fixed. We fixed the covariates (age, gender, levels of education and self-reported health) at their means.

Secondly, we evaluated possible two-way interactions between non-Hispanic Asian race/ethnicity and each independent variable in our model. Interaction terms are included into the model to explore whether care experience reported by Asians differ from others in the sample by age, gender, levels of education and reported health status. Non-significant interaction terms (*p* = 0.05) were excluded from the final model.

For the analyses of the 0–10 *rating of the doctor* and *would recommend doctor to friend/family* items, we initially considered estimating ordinal logistic regression models as a sensitivity analysis in addition to the OLS models. The Brant test was used to evaluate the proportionality assumption. The proportional odds assumption was not met for 16 out of 20 variables in the *0–10 rating of the doctor* model and 13 out of 20 variables in the *would recommend doctor to friend/family* model. Therefore, generalized models were used.

Positive response tendencies can be corrected by standard case-mix adjustments (i.e. for age, education) in a regression analysis. Extreme response tendencies, on the other hand, are not addressed by case-mix adjustments in the presence of skewed data. Weech-Maldonado et al. [20] recommended pooling responses at the lower *(0–6)* and top end *(9–10)* when examining racial/ethnic differences in CAHPS ratings of care. In this paper, we used this categorization approach (*0–6, 7–8, 9–10*) in a generalized logistic regression analysis.

## Results

Response rates for the survey are reported by 470 physician groups out of 769. The lowest reported response rate is 6%, the highest 97% and the median – 35%. Because these are self-reported by sponsors and could be biased, appropriate caution is warranted.

Substantive CAHPS survey questions are only asked of those for which they apply. Inappropriately missing values ranged from 1% to 6%. Means and standard deviations for the CAHPS items and composites are provided in Tables 3 and 4. Reports about care and global rating items are negatively skewed, with skewness ranging from − 0.93 to − 4.52. Intraclass correlations for the three factors ranged from 0.03 to 0.10 (*access to care* – 0.1, *physician communication and global ratings – 0.04, office staff helpfulness and courtesy – 0.03*) supporting individual-level factor analyses.

**Table 3:** Means and standard deviations for the five dependent variables in the CAHPS clinician and group adult visit survey 1.0

Composites	Total sample	Asian-Americans	Non-Hispanic Whites
**Access**			
*Mean*	78.69	68.76	79.89
*Std. Dev.*	23.99	26.74	23.09
*N*	263,737	5098	215,719
**Communication**			
*Mean*	94.15	93.11	94.47
*Std. Dev.*	14.69	15.22	14.16
*N*	265,783	5144	217,101
**Office**			
*Mean*	94.70	90.95	95.09
*Std. Dev.*	15.67	19.44	15.06
*N*	258,785	5091	213,493
**Rating a doctor**			
*Mean*	91.25	88.60	91.41
*Std. Dev.*	14.16	15.47	13.74
*N*	263,216	5089	215,535
**Recommending to friends/family**			
*Mean*	92.93	89.36	93.32
*Std. Dev.*	20.92	24.45	20.31
*N*	257,813	5056	212,384

All were scaled on a 0–100 possible range and the observed minimum and maximum were 0 and 100 for each variable

**Table 4:** Standardized factor loadings and standard errors for CAHPS clinician and group adult visit survey 1.0 items

Factor/Composite items	Factor loadings	Standard Error
Physician communication and global ratings		
*1 Explained things in a way that was easy to understand*	0.90	0.001
*2 Listened carefully*	0.96	0.001
*3 Gave easy to understand instructions*	0.89	0.001
*4 Was knowledgeable about medical history*	0.78	0.002
*5 Showed respect*	0.96	0.001
*6 Spent enough time*	0.88	0.001
*7 Rating a doctor*	0.84	0.001
*8 Recommending a doctor to friends/family*	0.92	0.001
Access		
*1 Timely appointment for urgent care*	0.88	0.002
*2 Timely appointment for check-up or routine care*	0.84	0.002
*3 Received answer to medical questions the same day, regular hours*	0.78	0.003
*4 Received answer to medical questions timely, after regular hours*	0.83	0.006
*5 Was seen within 15 min of appointment time*	0.57	0.002
Office staff		
*1 Was helpful*	0.98	0.003
*2 Treated with courtesy and respect*	0.94	0.003

Estimated correlations among factors were: Physician communication and global ratings with access (*r* = 0.60) and office staff (*r* = 0.46); Access with office staff (*r* = 0.51)

### Factor structure

The practical fit indices for the three-factor model were acceptable: CFI was 0.99 and RMSEA was 0.03. All factor loadings were statistically significant (*P* > 0.01) and above the 0.40 cut point (Table 4). The smallest loading was found for the item asking about how often a respondent was seen within 15 min of an appointment (0.57). None of the estimated correlations among factors were above the 0.80 threshold and they ranged from 0.46 to 0.60.

Single group CFA model outputs supported an acceptable model fit for both Asians and non-Hispanic Whites (Table 5). The model for Asians produced RMSEA (upper and lower limits of 90% confidence interval) = 0.037 (0.040; 0.034), CFI = 0.991 and TLI = 0.989 and for Whites = 0.031 (0.031; 0.030), CFI = 0.994. All factor loadings were statistically significant at *p* < 0.001 and greater than 0.50. While the loadings for Asians were uniformly higher than for Whites for *access to care* and *office staff courtesy and helpfulness* composites, the loadings for *physician communication and global ratings* did not differ between the two subgroups. The magnitude of difference in the standardized loadings between the two groups was the greatest for the item on timely response to a medical question after office hours (0.075; *p* < 0.01).

**Table 5:** Model fit indices for single group and multiple group confirmatory factor analytic models

	Free Parameters	Chi-Square Test of Model Fit	Degrees of Freedom	*P*	Root Mean Square Error of Approximation	Comparative Fit Index	Tucker-Lewis Index
Single group							
Asian Americans	54.00	702.08	87.00	0.00	0.037	0.991	0.989
Non-Hispanic Whites	54.00	17,993.25	87.00	0.00	0.031	0.994	0.992
Multiple Group, WLSMV^a^							
Configural	108.00	18,326.90	174.00	0.00	0.031	0.993	0.992
Metric	96.00	15,416.08	186.00	0.00	0.027	0.994	0.994
Scalar	63.00	15,885.50	219.00	0.00	0.025	0.994	0.995
Multiple Group, WLSMV^b^							
Configural	108.00	18,326.90	174.00	0.00	0.031	0.993	0.992
Metric	93.00	11,889.05	189.00	0.00	0.024	0.996	0.995
Scalar	57.00	14,248.89	225.00	0.00	0.024	0.995	0.995
Multiple Group, MLR^a^							
Configural	96.00	33,147.88	174.00	0.00	0.041	0.945	0.933
Metric	84.00	32,773.24	186.00	0.00	0.040	0.945	0.938
Scalar	72.00	33,976.08	198.00	0.00	0.039	0.943	0.940
Multiple Group, MLR^b^							
Configural	96.00	33,147.88	174.00	0.00	0.041	0.945	0.933
Metric	81.00	32,627.64	189.00	0.00	0.039	0.945	0.939
Scalar	66.00	34,865.43	204.00	0.00	0.039	0.942	0.940
	96.00	61,099.59	174.00	0.00	0.056	0.950	0.939
	84.00	61,359.40	186.00	0.00	0.054	0.950	0.943
	72.00	61,858.19	198.00	0.00	0.053	0.949	0.946
	96.00	61,099.59	174.00	0.00	0.056	0.950	0.939
	81.00	61,963.07	189.00	0.00	0.054	0.949	0.943
	66.00	63,750.85	204.00	0.00	0.053	0.948	0.946

*WLSMV* Weighted least squares, mean and variance adjusted, *MLR* Maximum likelihood robust, *ML* Maximum likelihood

^a^Factor means fixed to 0 in both groups and factor variances fixed to 1 in one group and freed in the second group

^b^Factor means fixed to 0 and factor variances fixed to 1 in both groups

### Measurement invariance

In the MG CFA analysis using WLSMV estimation, the fit indices for the configural invariance model were in the acceptable range – RMSEA (the upper and lower limit of 90% confidence interval) = 0.031 (0.031; 0.030), CFI = 0.993.

We conducted two versions of metric and scalar invariance testing using WLSMV estimation. Both versions of the tests showed an acceptable model fit (Table 5). The metric invariance model with factor means fixed to 0 and factor variances fixed to 1 in both groups produced a slightly better fit compared with the model with factor means fixed to 0 in both groups and factor variances fixed to 1 in one group and no constraints placed in the other group (RMSEA = 0.024 vs. 0.027, CFI = 0.996 vs. 0.994). Neither of the scalar invariance models produced consistently better fit indices (RMSEA = 0.024 vs. 0.025, CFI = 0.995 vs. 0.994). Both MLR and ML models supported metric invariance when using approximate fit indices.

### Racial-ethnic disparities in patient experiences

Online Supplementary Material Table S1 presents recycled predictions (main effects and interaction terms models). Asian Americans reported the worst access (*predicted score*: Asian Americans (72.10), non-Hispanic Whites (79.10), African American (79.01), Hispanic (77.78) and Other (77.97)) and lowest (worse experiences) scores (*predicted score*: Asian Americans (92.74), non-Hispanic Whites (94.85), African American (95.26), Hispanic (94.55) and Other (93.60)) on the office staff courtesy and helpfulness measure of all five racial/ethnic groups. Asian Americans also reported worse scores on rating their doctors and were also the least likely to recommend their doctors to family and friends of all five racial/ethnic groups. There were no significant differences between Asian Americans and non-Hispanic Whites on physician communication. The “other” racial/ethnic group reported the worst physician communication.

We were unable to explore regional variations in reports and ratings among Asian Americans in our main model due to collinearity between the practice site and region. We ran a secondary model where we replaced the practice site with the region and found that Asian Americans from the Northeast report better experience than Asian Americans from the West. Asian Americans in the South rate care worse on most measures than Asian Americans in the West, Midwest and Northeast.

Several interactions between Asian American race/ethnicity and gender, age, education, and health were significant. For example, Asian Americans who rate their health as *excellent* reported better experience than Asian Americans with other self-reported health states. Asian Americans in the 45–54 age group reported worse access to care compared to Asian Americans of other ages. Asian Americans with less than high school education had the worst access among Asian Americans of various education levels. However, the interactions were not in a consistent direction.

The findings from the generalized ordinal logistic models for the 0–10 *rating of the doctor* and *would recommend doctor to friend/family* items were in general consistent with the OLS model results and are presented in the Online Supplemental Material, Table S2.

## Discussion

The categorical confirmatory factor analysis showed that the three factor structure fits well in our dataset and in line with the findings from the continuous factor analysis conducted by Dyer et al. [12]. All the items loaded significantly on to the respective factors. The lowest loadings were observed for the item on wait times at the doctor’s office. This item was shown to load weakly on access to care related factors in other CAHPS surveys as well [21, 22]. Our study provides support for measurement invariance between Asians and Whites in the CG-CAHPS Adult Visit Survey 1.0 measures of *access to care*, *physician communication and global ratings*, and *office staff courtesy and helpfulness*. The criteria for both metric and scalar invariance were met, suggesting that the mean differences reported in the CG-CAHPS Adult Visit Survey 1.0 between these two groups were likely to be due to differences in care experiences. Asian Americans reported the worst scores on *access to care, office staff courtesy and helpfulness*, *rating of their doctors* and were the least likely *to recommend their doctors to family and friends* of the all five racial/ethnic groups. Given support for measurement invariance in our analyses, we did not explore differential item functioning further [23, 24].

Our study makes several important contributions to the literature. Earlier studies analyzed Asian Americans and Pacific Islanders together; a sufficiently large sample of Asian Americans in our dataset allowed us to analyze Asian Americans separately from Pacific Islanders. Our findings also show that regional variations in patient experiences among Asian Americans exist. Underlying reasons for regional variations among Asian Americans in CAHPS surveys are little studied and require further research.

Racial-ethnic disparities can be driven by differential access or selection into plans or providers of differing quality. However, several studies report that “within provider” differences account for the significant share of disparities between Asian Americans and non-Hispanic Whites. In our study, we control for the “between providers” effects by including in our model provider identifications.

While several studies have evaluated the factor structure of CAHPS surveys, only Dyer et al. [12] have evaluated the CG-CAHPS Adult Visit survey 1.0 and this was done using factor analysis that assumed continuous items and within only a subset of the sample. We conducted MG CFA using three estimation approaches: theta parametrization and WLSMV estimation (used for categorical variables), MLR and ML estimation. Our analysis also found that chi square values may not be directly comparable across models in MG CFA analyses when MLR estimation is used with categorical data. Further research will be needed to explore whether this is supported in other datasets.

Our study has several limitations. Although the sample analyzed represented respondents with various racial/ethnic and demographic backgrounds, the respondents were predominantly White and educated. In addition, response rates were not available in the dataset. CAHPS adult surveys in samples other than Medicare tend to yield response rates below 40%. If non-responders differ in how they interpret and respond to survey questions, the study results may not generalize to patients in care more generally. Moreover, the dataset did not include information about insurance and that could explain some of the observed differences. Furthermore, we did not have information about Asian subgroups, English proficiency, nativity, and time in the U.S. Asians are a diverse racial/ethnic group comprising of various subgroups (e.g., Chinese, Japanese, Vietnamese). The subgroups may have different patient care experiences and may differ in how they interpret and respond to CAHPS survey items. Those with limited English proficiency, and in the U.S for a short time may have worse care experiences due to difficulties in communication or limited experience in navigating the complex US healthcare system. They are also less likely to respond to survey questions, thus possibly biasing the study findings. Asians who marked Hispanic origin (*n* = 135) were treated as Hispanics in this analysis. Future studies should explore reported differences and measurement invariance among various Asian subgroups, including Hispanic Asians.

In conclusion, the validity of racial/ethnic comparisons of reports of patient experiences is critical to informing quality improvement initiatives and policy decisions. The findings of this study support the use of the CG-CAHPS Adult Visit Survey 1.0 measures in comparisons of patient perceptions of care across Asians and Whites in the US. The differences in reports and ratings of care reported between Asians and Whites in the CG-CAHPS Adult Visit Survey 1.0 data are likely due to differences in care experiences. Future studies are required to explore the underlying reasons for the racial-ethnic differences in physician communication, access to care and office staff support. These studies should aim to inform care providers and payers about underlying reasons for differences and help to tailor quality improvement initiatives to address racial-ethnic disparities in care.

## Supplementary Information

**Additional file 1: Online Supplemental Material Table 1.** Recycled predictions from main effects only model and the model with the interaction terms. **Online Supplemental Material Table 2.** Generalized ordinal logistic regression. **Online Supplemental Material Figure 1.** Three-factor model.

## Data Availability

The dataset analyzed during the current study was obtained from the Agency for Healthcare Research and Quality: https://cahpsdatabase.ahrq.gov/files/CG/CAHPS_Database_Overview.pdf.

## Abbreviations

CAHPS: Consumer Assessment of Healthcare Providers and Systems
CG-CAHPS: Clinical and Group Consumer Assessment of Healthcare Providers and Systems
RMSEA: Root mean squared error of approximation
CFA: Confirmatory factor analysis
CFI: Comparative fit index
MG CFA: Multiple group confirmatory factor analysis
OLS: Ordinary least squares
WLSMV: Weighted least square mean and variance adjusted estimation
