# Supplementary material for: Consumer Assessment of Healthcare Providers and Systems (CAHPS®) survey of experiences with ambulatory healthcare for Asians and non-Hispanic Whites in the United States
Source: J Patient Rep Outcomes. 2021 Mar 24;5:29. doi: 10.1186/s41687-021-00303-3 (PMC7990982; doi:10.1186/s41687-021-00303-3)
Supplement: Supplementary file 1 — Additional file 1: Online Supplemental Material Table 1. Recycled predictions from main effects only model and the model with the interaction terms. Online Supplemental Material Table 2. Generalized ordinal logistic regression. Online Supplemental Material Figure 1. Three-factor model. [file 41687_2021_303_MOESM1_ESM.zip › Online Supplemental Material_Tables.docx]

**Online Supplemental Material Table 1:** *Recycled predictions from main effects only model and the model with the interaction terms*

|  | Access | | Communication | | Office Staff | | Overall Rating | | Would Recommend | |
| --- | --- | --- | --- | --- | --- | --- | --- | --- | --- | --- |
|  | Margin | SE | Margin | SE | Margin | SE | Margin | SE | Margin | SE |
| Predicted scores from the main effects model | | | | | | | | | | |
| **Race/ethnicity** |  |  |  |  |  |  |  |  |  |  |
| White | 79.10 | 0.03 | 94.31 | 0.02 | 94.85 | 0.02 | 91.30 | 0.02 | 93.11 | 0.02 |
| Asian American | 72.38 | 0.43 | 94.03 | 0.23 | 92.74 | 0.32 | 89.79 | 0.27 | 91.01 | 0.37 |
| Hispanic | 77.78 | 0.36 | 94.12 | 0.22 | 94.55 | 0.21 | 91.91 | 0.19 | 93.43 | 0.28 |
| African American | 79.01 | 0.45 | 94.55 | 0.25 | 95.26 | 0.19 | 91.95 | 0.30 | 93.58 | 0.40 |
| Other | 77.97 | 0.20 | 93.03 | 0.14 | 93.60 | 0.17 | 90.78 | 0.13 | 91.68 | 0.20 |
| Predicted Scores from the model with interaction terms | | | | | | | | | | |
| **Asian x Health (Poor)** | 72.38 |  | 94.03 |  | 92.04 |  | 89.79 |  | 91.01 |  |
| Asian x Fair | 72.38 |  | 94.03 |  | 92.04 |  | 89.79 |  | 91.01 |  |
| Asian x Good | 72.38 |  | 94.03 |  | 92.04 |  | 89.79 |  | 91.01 |  |
| Asian x Very Good | 72.38 |  | 94.03 |  | 92.04 |  | 89.79 |  | 91.01 |  |
| Asian x Excellent | 72.38 |  | 94.03 |  | 93.79 |  | 89.79 |  | 91.01 |  |
| **Asian x Age (18-24)** | 72.76 |  | 94.03 |  | 92.74 |  | 89.79 |  | 91.01 |  |
| Asian x 25-34 | 72.76 |  | 94.03 |  | 92.74 |  | 89.79 |  | 91.01 |  |
| Asian x 35-44 | 72.76 |  | 94.03 |  | 92.74 |  | 89.79 |  | 91.01 |  |
| Asian x 45-54 | 71.25 |  | 94.03 |  | 92.74 |  | 89.79 |  | 91.01 |  |
| Asian x 55-64 | 72.76 |  | 94.03 |  | 92.74 |  | 89.79 |  | 91.01 |  |
| Asian x 65-74 | 72.76 |  | 94.03 |  | 92.74 |  | 89.79 |  | 91.01 |  |
| Asian x 75 & over | 72.76 |  | 94.03 |  | 92.74 |  | 89.79 |  | 91.01 |  |
| **Asian x Education (8th Grade)** | 74.61 |  | 94.03 |  | 92.74 |  | 89.79 |  | 91.01 |  |
| Asian x < High School | 70.44 |  | 94.03 |  | 92.74 |  | 89.79 |  | 91.01 |  |
| Asian x High School | 72.10 |  | 94.03 |  | 92.74 |  | 89.79 |  | 91.01 |  |
| Asian x < College | 74.61 |  | 94.03 |  | 92.74 |  | 89.79 |  | 91.01 |  |
| Asian x Some College | 74.61 |  | 94.03 |  | 92.74 |  | 89.79 |  | 91.01 |  |
| Asian x College Graduate | 74.61 |  | 94.03 |  | 92.74 |  | 89.79 |  | 91.01 |  |

**Online Supplemental Material Table 2:** *Generalized ordinal logistic regression*

|  | **Rating (1)**  **(χ^2^-0.00)** | | | **Rating (2)**  **(χ^2^-0.00)** | | | **Recommending (1)**  **(χ^2^-0.00)** | | | **Recommending (2)**  **(χ^2^-0.00)** | | |
| --- | --- | --- | --- | --- | --- | --- | --- | --- | --- | --- | --- | --- |
|  | Coef. | . SE. | z | Coef. | SE. | z | Coef. | SE. | z | Coef. | SE. | z |
| **Race-ethnicity (White)** |  |  |  |  |  |  |  |  |  |  |  |  |
| Asian American | **-0.19** | **0.07** | **-2.80** | **-0.32** | **0.05** | **-6.99** | -0.15 | 0.09 | -1.63 | **-0.39** | **0.04** | **-8.94** |
| Hispanic | **-0.27** | **0.06** | **-4.52** | -0.04 | 0.04 | -0.93 | **-0.26** | **0.06** | **-4.42** | **-0.26** | **0.06** | **-4.42** |
| African American | 0.07 | 0.04 | 1.62 | 0.07 | 0.04 | 1.62 | 0.01 | 0.05 | 0.29 | 0.01 | 0.05 | 0.29 |
| Other | **-0.20** | **0.04** | **-5.38** | **-0.05** | **0.02** | **-2.23** | **-0.34** | **0.05** | **-6.98** | **-0.21** | **0.03** | **-7.15** |
| **Health (Poor)** |  |  |  |  |  |  |  |  |  |  |  |  |
| Fair health | **0.31** | **0.04** | **7.60** | **0.06** | **0.03** | **2.18** | **0.58** | **0.05** | **10.69** | **0.18** | **0.03** | **5.45** |
| Good health | **0.70** | **0.04** | **15.79** | **0.22** | **0.03** | **7.10** | **1.02** | **0.06** | **18.46** | **0.41** | **0.04** | **10.71** |
| Very good health | **1.17** | **0.05** | **23.82** | **0.50** | **0.03** | **15.19** | **1.48** | **0.06** | **26.01** | **0.73** | **0.04** | **18.26** |
| Excellent health | **1.49** | **0.06** | **25.99** | **1.01** | **0.04** | **27.74** | **1.70** | **0.07** | **24.41** | **1.20** | **0.04** | **27.38** |
| **Age (18-24)** |  |  |  |  |  |  |  |  |  |  |  |  |
| Age 25-34 | **0.18** | **0.03** | **6.37** | **0.18** | **0.03** | **6.37** | 0.04 | 0.05 | 0.92 | **0.11** | **0.04** | **3.03** |
| Age 35-44 | **0.43** | **0.03** | **14.46** | **0.43** | **0.03** | **14.46** | **0.37** | **0.04** | **9.55** | **0.37** | **0.04** | **9.55** |
| Age 45-54 | **0.66** | **0.04** | **17.21** | **0.59** | **0.03** | **19.15** | **0.56** | **0.04** | **14.57** | **0.56** | **0.04** | **14.57** |
| Age 55-64 | **0.96** | **0.04** | **22.79** | **0.81** | **0.03** | **24.40** | **0.93** | **0.05** | **18.63** | **0.78** | **0.04** | **19.44** |
| Age 65-74 | **1.23** | **0.04** | **28.84** | **1.03** | **0.03** | **31.87** | **1.22** | **0.06** | **22.03** | **1.00** | **0.04** | **25.92** |
| Age 75 and over | **1.22** | **0.05** | **25.67** | **1.02** | **0.04** | **27.72** | **1.34** | **0.06** | **21.02** | **0.98** | **0.04** | **22.24** |
| **Education (8th Grade)** |  |  |  |  |  |  |  |  |  |  |  |  |
| < High School | 0.06 | 0.05 | 1.29 | 0.06 | 0.05 | 1.29 | 0.01 | 0.07 | 0.11 | 0.01 | 0.07 | 0.11 |
| High School | 0.08 | 0.05 | 1.66 | **-0.09** | **0.04** | **-2.29** | -0.10 | 0.06 | -1.81 | -0.10 | 0.06 | -1.81 |
| < College | 0.06 | 0.05 | 1.17 | **-0.12** | **0.04** | **-2.82** | **-0.25** | **0.07** | **-3.64** | **-0.13** | **0.06** | **-2.19** |
| Some college | 0.07 | 0.06 | 1.30 | **-0.30** | **0.04** | **-7.21** | **-0.24** | **0.06** | **-4.16** | **-0.24** | **0.06** | **-4.16** |
| College Graduate | 0.03 | 0.06 | 0.47 | **-0.29** | **0.04** | **-6.51** | **-0.38** | **0.07** | **-5.33** | **-0.27** | **0.06** | **-4.41** |
| **Gender** |  |  |  |  |  |  |  |  |  |  |  |  |
| Male | **0.23** | **0.03** | **9.10** | 0.00 | 0.02 | -0.17 | **0.17** | **0.03** | **5.12** | **0.10** | **0.02** | **4.90** |
| **Constant** | **1.29** | **0.07** | **17.52** | **0.35** | **0.06** | **5.74** | **1.95** | **0.08** | **23.25** | **1.00** | **0.09** | **11.56** |

**Note: (1) = Category 1 versus 2 and 3; (2) Category 1 and 2 versus 3.**

**Online Supplemental Material Figure 1**

*Three-factor model*

Image file: figure1.tiff
