# Supplementary figures and images for: Consumer Assessment of Healthcare Providers and Systems (CAHPS®) survey of experiences with ambulatory healthcare for Asians and non-Hispanic Whites in the United States
Source: J Patient Rep Outcomes. 2021 Mar 24;5:29. doi: 10.1186/s41687-021-00303-3 (PMC7990982; doi:10.1186/s41687-021-00303-3)

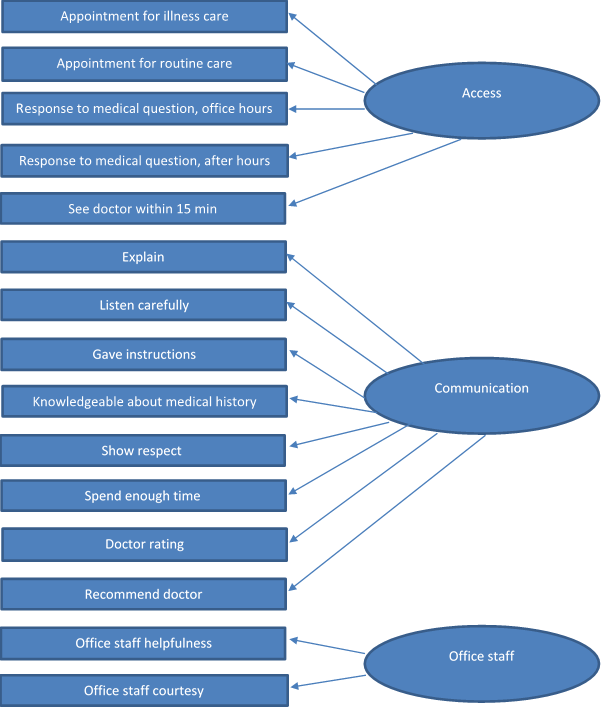

Supplement: Supplementary file 1 — Additional file 1: Online Supplemental Material Table 1. Recycled predictions from main effects only model and the model with the interaction terms. Online Supplemental Material Table 2. Generalized ordinal logistic regression. Online Supplemental Material Figure 1. Three-factor model. [file 41687_2021_303_MOESM1_ESM.zip › Figure1.tif]
